# Wisdom from the past

**Published:** 1971-10

**Authors:** 


### Wisdom from the Past

### SEVENTY-FIVE YEARS AGO

### PROGRESS OF THE MEDICAL SCIENCES

**Medicine.** The discovery at Bath of an organism found almost invariably in joints affected by rheumatoid arthritis* is one of high importance, and calls for our hearty congratulations. The successful reproduction of the disease by inoculation, or its relief by an antitoxin prepared from the growth, will be eagerly looked for as the crowning proof in a laborious investigation.

Many observers have of late sought in vain for a similar cause for acute rheumatism, which presents many analogies to septic diseases; but though Leyden has cultivated a microbe from rheumatic endocarditis, still that lesion is caused in so many different pathological circumstances that little importance can be attached to it. In speaking of this lesion we may refer to the practical results in preventing it recorded by Caton. The proportion of heart troubles can, he thinks, be greatly reduced by his method. He not only insists on absolute rest with flannel bedclothes and salicylates, but as soon as the slightest blurring of the heart-sounds occurs, he applies a small blister over the apex of the heart, and, as the effects of that pass away, another close to it, and so on, until with absolute rest, and the addition of diodides to the salicylates the murmur disappears, as it generally does if this treatment is persevered with long enough.

*Reported in detail, page 123 of same number.

**—Vol. 14, p. 155 (1896)**

### FIFTY YEARS AGO

**University of Bristol. Postgraduate Studies.** The work of the Medical Postgraduate Committee is steadily extending, and courses of medical and surgical demonstrations are being held at Trowbridge, Swindon and Dorchester. Enquiries are being made from other districts, and there is every prospect of a wide extension of postgraduate work throughout the West of England.

The Director has received promises of demonstrations from many members of the teaching staff of the University, and synopses of these lectures are now printed and can be forwarded to prospective centres from which inquiries are received. Medical men forming provincial classes will thus have a wide range of choice of subject.

The lectures are generally in abeyance during December and January, but during next spring not only will these classes be resumed in Bristol and in the country, but it is hoped that a week may be devoted in Bristol to postgraduate study in one or other of the special departments.

The Clinical Assistantships available for one month, or thirty attendances convenient to the practitioner, have attracted a number of practitioners, and demonstrated the wide facilities offered by the University and the clinical institutions.

**Royal Infirmary.** At the half-yearly Board of Governors held on October 13th the announcement of the resignation of the President, Mr. H. H. Wills, was received with the greatest regret. Ill-health has compelled Mr. Wills to take this course. During his tenure of office his interest and energy on behalf of the institution have been invaluable, whilst his generous gift of £100,000 in the early part of this year has made a substantial difference to the financial difficulties with which the Infirmary, like all other voluntary hospitals, was faced.

The ALMONER'S REPORT presented at the same meeting contains a remark which arrests our attention. The Almoner states that of all the patients admitted to the Institution only one in five is able to pay the very modest contribution for maintenance which is asked for. From this observation, based on most careful enquiry by an experienced officer well qualified to judge, but one conclusion can be drawn, viz. that the payments by patients will not materially reduce the expenses of the Infirmary so long as it continues to provide for the wants of the really necessitous persons for whom the charity was founded and for whom it has hitherto been maintained. The Governors are now confronted with the serious question whether sufficient voluntary contributions will be forthcoming from the more affluent members of the community, or whether they will be compelled to close their beds to the poor and divert their institution from its original purpose, admitting principally or solely those who can pay for admission. It would be intolerable if "ability to pay" should be the passport into our voluntary hospitals. The public, we venture to think, is unaware of the amount of distress in our midst. There is an impression abroad that wages are high and that all classes enjoy a fair measure of prosperity so that the claims of charity are less pressing. The report shows how erroneous this impression is. Funds are urgently needed, and no scheme for payment by patients is going to solve the difficulty of those who through ill-health have fallen into poverty. Unless voluntary contributions are more freely forthcoming the hospitals of our city will not be able to continue their mission for those whose needs are greatest. Hard times are at hand, when it becomes the more incumbent on us to show unwearied mercifulness touching the care of the poor. We must each and all unlock the treasury of our charity to its fullest extent, so that after the example of generations past we may discharge our just debts of benevolence.

**—Editorial Notes. Vol. 38, pp. 121-3 (1921)**

